# Unraveling the Impact of Travel on Circadian Rhythm and Crafting Optimal Management Approaches: A Systematic Review

**DOI:** 10.7759/cureus.71316

**Published:** 2024-10-12

**Authors:** Osman Ahmed, Amir T Ibrahiam, Zahraa M Al-Qassab, Vaishnavi Kannan, Najeeb Ullah, Sunitha Geddada, Sondos T Nassar

**Affiliations:** 1 Family Medicine, California Institute of Behavioral Neurosciences and Psychology, Fairfield, USA; 2 Internal Medicine, California Institute of Behavioral Neurosciences and Psychology, Fairfield, USA; 3 General Surgery Research, California Institute of Behavioral Neurosciences and Psychology, Fairfield, USA; 4 General Surgery, California Institute of Behavioral Neurosciences and Psychology, Fairfield, USA; 5 Medicine and Surgery, California Institute of Behavioral Neurosciences and Psychology, Fairfield, USA

**Keywords:** chronotherapy, circadian misalignment interventions, circadian rhythm, jet lag management, travel-induced circadian disruption

## Abstract

Circadian rhythms, which regulate essential physiological and behavioral processes, are crucial for maintaining overall health and well-being. However, disruptions to these rhythms, particularly due to trans-meridian travel and shift work, can lead to significant health issues, including jet lag, metabolic imbalances, and neuropsychiatric disorders. In this systematic review, conducted in line with Preferred Reporting Items for Systematic Reviews and Metanalysis (PRISMA) 2020 guidelines, we explore the effectiveness of various interventions aimed at realigning circadian rhythms disrupted by such factors. Focusing on studies published between 2020 and 2024, our search spanned databases like PubMed, Google Scholar, PubMed Central (PMC), and ScienceDirect, emphasizing randomized controlled trials (RCTs), observational studies, and comprehensive reviews. A rigorous quality assessment using the AMSTAR tool for systematic reviews and the SANRA checklist for narrative reviews was employed. Out of an initial 2153 titles, 23 high-quality studies were identified and analyzed. The findings reveal that interventions such as melatonin supplementation, personalized light exposure, and chrono-modulation can significantly improve sleep quality, reduce jet lag symptoms, and promote better health outcomes. This review highlights the critical nature of circadian alignment in preventing serious health issues, such as mental health disorders, metabolic syndromes, and even cancer. It advocates for a personalized, integrative approach that combines melatonin, light therapy, time-restricted eating, exercise, and alternative therapies like electroacupuncture. Such strategies not only facilitate smoother transitions across time zones but also contribute to overall health and resilience. Future studies should aim to assess the long-term benefits and practical applications of these interventions in broader populations.

## Introduction and background

"Time waits for no one, and neither do the consequences of disrupting our internal clocks." The rapid increase in international travel has led to a growing demand for long and ultra-long-haul flights, which in turn raises the risk of various physiological and psychological symptoms by disrupting our biological clocks [1]. Airline crew members and passengers often experience travel fatigue, jet lag, edema, deep vein thrombosis (DVT), and an increased susceptibility to illnesses such as colds. Recognizing this as a growing concern, numerous studies, including those by Francy Cruz-Sanabria et al., have examined the use of exogenous melatonin to understand its mechanisms and sleep-promoting effects in combating jet lag syndrome [2].

Jet lag syndrome occurs when transmeridian travel disrupts the body's circadian rhythm. Circadian rhythms, derived from the Latin "circa" (about) and "diem" (day), are endogenous oscillations that operate on an approximately 24-hour cycle. Any disruption, dysregulation, or factor that negatively impacts these rhythms is known as circadian disruption, which can be influenced by travel, shift work, or mistimed eating [3]. Jet lag specifically results from a time zone differential, which is affected by the direction and distance of travel, the duration of the flight, and the number of time zones crossed. When crossing three or more time zones, whether transmeridian or trans-lateral, the circadian system cannot immediately adapt to the new time zone. This leads to internal desynchrony of the circadian system, disrupting sleep homeostasis. In such cases, the circadian system remains aligned with the departure time zone rather than adjusting to the arrival time, resulting in the onset of jet lag syndrome [4]. 

Understanding the physiology of jet lag syndrome is crucial for comprehending the mechanisms behind interventions such as chronotherapy, and exogenous melatonin, and their impact on chronic pathologies like diabetes, depression, or cancer, which are influenced by the temporal misalignment of endogenous circadian oscillations. These interventions aim to restore circadian alignment and alleviate the symptoms of jet lag [5,6]. Melatonin plays a significant role in jet lag syndrome as it helps in regulating sleep. As suggested by J. J. Poza et al., the frequency of poor-quality sleep is inversely related to melatonin levels in an individual, indicating melatonin deficiency can lead to sleep disorders in older adults [7]. In addition to melatonin, appropriate meal timing, composition, and nutrients can also help with circadian dysregulation which is referred to as chrononutrition [8]. Light also plays a very important role in regulating circadian rhythm, a process known as chrono-phototherapy. It is the most dominant environmental factor responsible for synchronizing circadian rhythms [9]. 

Circadian rhythms regulate various processes at molecular, cellular, organ, and systemic levels over a 24-hour cycle. The circadian rhythm requires daily adjustments to align with the external environment, as the human body's internal clock operates on a slightly longer than 24-hour cycle. This alignment is achieved through the central pacemaker located in the suprachiasmatic nucleus (SCN) of the anterior hypothalamus [10,11]. Despite numerous studies focusing on individual interventions like melatonin or light therapy, there is a lack of comprehensive reviews that synthesize these varied strategies together, highlighting the novelty of our approach in evaluating the collective and integrative management of travel-induced circadian disruptions. This review seeks to explore the effectiveness of various interventions, including chronotherapy, melatonin supplementation, chrono nutrition, the concept of sleep hygiene, chrono-phototherapy, chrono-exercise, electroacupuncture, modulating casein kinases (CKs), modulating REV-ERBs (nuclear receptors) and others in addressing jet lag syndrome. By exploring how these interventions work to restore circadian alignment, this review will highlight their potential role in reducing the long-term health consequences of circadian disruption, such as metabolic, neurological, mental health, and oncological pathologies. By understanding these mechanisms, we can better inform therapies that not only alleviate the immediate symptoms of circadian disruption, such as jet lag but also mitigate its long-term health impacts. This review aims to explore how these targeted interventions can be optimized to address the increasing prevalence of circadian rhythm disturbances in modern life. 

## Review

Overview

Methods

The guidelines of Preferred Reporting Items for Systematic Reviews and Metanalysis (PRISMA) 2020 are used in this study [12]. 

Eligibility Criteria

This systematic review studies the impact of travel on circadian rhythm and evaluates the effectiveness of sleep-wake cycle manipulation through various medical and non-medical interventions. The review question was formulated using the PICO framework used in systematic reviews: participants (P), intervention (I), and outcome (O). The participants include transmeridian travelers. The intervention includes sleep-wake cycle manipulation and medical and non-medical management, and the outcome is the reversal of circadian misalignment.

The inclusion criteria for this review include studies published between 2020 and 2024, full free-text articles available in English, and studies involving human participants. Eligible study designs include literature reviews, Gray literature, randomized controlled trials (RCTs), observational studies, case reports, systematic reviews, and meta-analyses. Studies must focus on the impact of sleep-wake cycle manipulation or chronotherapy on reversing circadian misalignment in transmeridian travelers.

Exclusion criteria include articles published before 2020, papers not available in English, and editorials. By following these criteria, this review aims to offer a thorough synthesis of existing evidence on the effectiveness of manipulating the sleep-wake cycle to address circadian misalignment in individuals traveling across multiple time zones.

Database and Search Strategy Involved in the Study

A comprehensive and systematic search was conducted using the following databases: Google Scholar, PubMed, PubMed Central (PMC), and ScienceDirect. The most recent search was completed in January 2024. The search utilized key terms such as "Circadian rhythm," "Travel," and "Prevention and management." In PubMed, the search was further refined using the Medical Subject Heading (MeSH) strategy. Comprehensive details of the databases and the specific search strategies used are provided in Table 1.

**Table 1:** Detailed description of databases' search terms and results PMC: PubMed Central

Databases	Keywords	Search strategy	Number of articles before filters	Filters	Search result
PubMed	Circadian rhythm OR biologic clock OR jet lag OR biologic rhythm Travel OR movement OR trip OR fly OR air travel Chronotherapy OR circadian rhythm therapy OR biologic rhythm modulation OR sleep wake cycle manipulation OR chronobiotic OR melatoninergic drugs	( "Jet Lag Syndrome/classification"[Mesh] OR "Jet Lag Syndrome/complications"[Mesh] OR "Jet Lag Syndrome/diagnosis"[Mesh] OR "Jet Lag Syndrome/diagnostic imaging"[Mesh] OR "Jet Lag Syndrome/mortality"[Mesh] OR "Jet Lag Syndrome/pathology"[Mesh] OR "Jet Lag Syndrome/physiopathology"[Mesh] ) ( "Air Travel/classification"[Mesh] OR "Air Travel/psychology"[Mesh] ) ( "Jet Lag Syndrome/diet therapy"[Mesh] OR "Jet Lag Syndrome/drug therapy"[Mesh] OR "Jet Lag Syndrome/prevention and control"[Mesh] OR "Jet Lag Syndrome/psychology"[Mesh] OR "Jet Lag Syndrome/radiotherapy"[Mesh] OR "Jet Lag Syndrome/rehabilitation"[Mesh] OR "Jet Lag Syndrome/surgery"[Mesh] OR "Jet Lag Syndrome/therapy"[Mesh] )	24,371	4 years (2020-2024), full free-texts, English, Human	1,036
Google Scholar	Circadian rhythm OR biologic clock OR jet lag AND Chronotherapy OR biologic rhythm modulation OR sleep wake cycle manipulation OR melatoninergic drugs	"Circadian rhythm" OR "biologic clock" OR "jet lag" AND “Travel” OR “movement” OR “fly” AND "Chronotherapy" OR "biologic rhythm modulation" OR "sleep wake cycle manipulation" OR "melatoninergic drugs"	3,280	4 years cut off	1,020
Science Direct	Jet lag AND travel AND chronotherapy	Jet lag AND Travel AND Chronotherapy	145	4 years cut off	31
PMC	Jet lag AND travel AND chronotherapy	Jet lag AND travel AND chronotherapy	129	4 years cut off	66

All the references mentioned above were organized and alphabetized using EndNote, with duplicate entries removed through both EndNote’s automatic process and manual review. The remaining records were screened by reviewing their titles followed by reading the abstracts, and irrelevant studies were excluded. Full-text articles from the relevant studies were then obtained and evaluated using an appropriate quality appraisal tool as per the study type to reduce the risk of bias in this research.

Risk of Bias in Individual Studies

Throughout the study selection and quality assessment processes, no conflicts or discrepancies arose. For quality assessment and risk of bias evaluation, the full articles retrieved were assessed using appropriate tools depending on the study type. Systematic reviews were evaluated as per the Assessment of Multiple Systematic Reviews (AMSTAR) tool, while literature reviews were assessed with the Scale for the Assessment of Narrative Review Articles (SANRA) checklist. Each assessment tool has its criteria and passing scores, and a 70% score was required for an article to be accepted. Detailed information on these tools and their application can be found in Table 2.

**Table 2:** AMSTAR for systematic reviews and SANRA checklist for literature reviews SANRA: Scale for the Assessment of Narrative Review Articles; AMSTAR: Assessment of Multiple Systematic Reviews

Quality assessment tool	Type of study	Items and their characteristics	Total score	Accepted score	Accepted studies	Number of accepted studies
AMSTAR 2 [13]	Systematic review	16 Domains: 1. Did the research questions and inclusion criteria for the review include the components of PICO? 2. Did the report of the review contain an explicit statement that the review methods were established prior to the conduct of the review and did the report justify any significant deviations from the protocol? 3. Did the review authors explain their selection of the study designs for inclusion in the review? 4. Did the review authors use a comprehensive literature search strategy? 5. Did the review authors perform study selection in duplicate? 6. Did the review authors perform data extraction in duplicate? 7. Did the review authors provide a list of excluded studies and justify the exclusions? 8. Did the review authors describe the included studies in adequate detail? 9. Did the review authors use a satisfactory technique for assessing the risk of bias (RoB) in individual studies that were included in the review? 10. Did the review authors report on the sources of funding for the studies included in the review? 11. If meta-analysis was performed did the review authors use appropriate methods for statistical combination of results? 12. If meta-analysis was performed, did the review authors assess the potential impact of risk of bias in individual studies on the results of the meta-analysis or other evidence synthesis? 13. Did the review authors account for RoB in individual studies when interpreting/discussing the results of the review? 14. Did the review authors provide a satisfactory explanation for, and discussion of, any heterogeneity observed in the results of the review? 15. If they performed quantitative synthesis did the review authors carry out an adequate investigation of publication bias (small study bias) and discuss its likely impact on the results of the review? 16. Did the review authors report any potential sources of conflict of interest, including any funding they received for conducting the review? Yes	High/moderate/low/critically low	High	Chan V, Wang L et al. [1] and Cruz-Sanabri et al. [2]	2
SANRA 2 [14]	Literature review	Six items: (1) justification of the article’s importance for the readership, (2) statement of concrete/specific aims or formulation of questions, (3) description of the literature search, (4) referencing, (5) scientific reasoning, and (6) appropriate presentation of data, scored as 0, 1, and 2	12	9	Fishbein Abet al. [3], Russell G. Foster et al. Foster RG. Sleep et al. [4], Janse van Rensburg DC et al. [5], Lee Y et al. [6], Li Y et al. [7], Poza JJ et al. [8], Senesi P et al. [9], Spaleniak W et al. [10], Sun SY et al. [11], Drăgoi CM et al. [15], Samanta S et al. [16], Roenneberg T et al. [17], Moreno CRC et al. [18], Healy KL et al [19], Lee Y et al. [20], Aykan U et al. [21], Vasey C et al. [22], and Yuan M et al. [23]	21

Results

**Figure 1:**
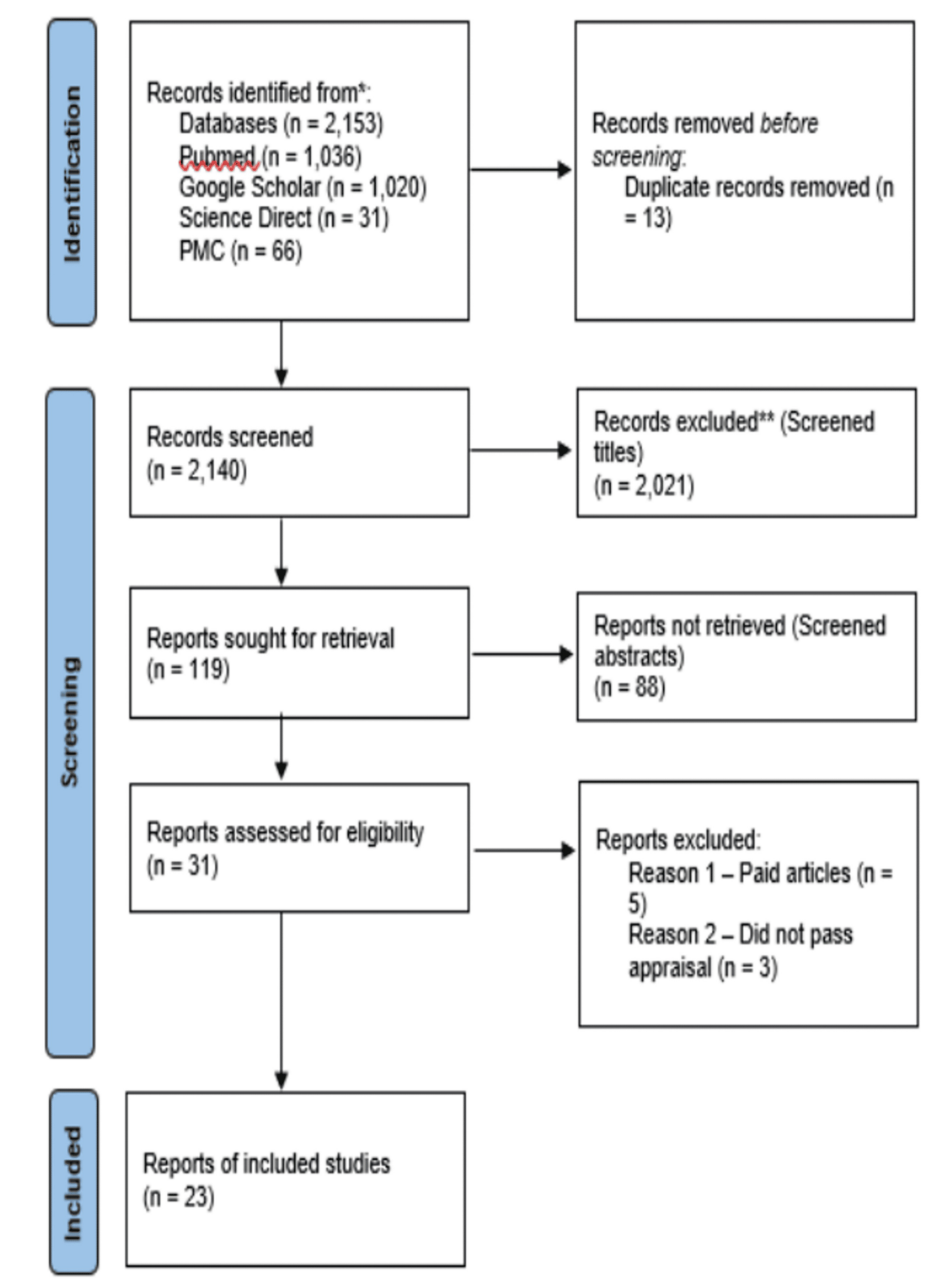
Flowchart of the study search selection PRISMA 2020 statement [12].

At the beginning of the database search, 2,153 potentially relevant titles were identified. After removing 13 duplicates, 2,140 records remained. During the next phase, 2,021 articles were screened by their titles and abstracts based on PIO elements and eligibility criteria of this review, which excluded 119 articles for full-text retrieval. The first author conducted a quality assessment of the retrieved reports, which was reviewed and agreed upon by the second and third authors. This process resulted in 23 studies passing the appraisal assessment and being included in the review. These included two systematic reviews and 19 literature reviews. A flow diagram illustrating the screening process and study selection is presented in Figure 1.

Table 3 and Table 4 show how each study was evaluated according to the corresponding study type and the result of the evaluation process.

**Table 3:** Results of the AMSTAR 2 assessment tool for systematic reviews by review authors AMSTAR: Assessment of Multiple Systematic Reviews Passing score: High

Domain	First author	
	Chan V, Wang L et al. [1]	Cruz-Sanabri et al. [2]
1. Did the research questions and inclusion criteria for the review include the components of PICO?	Yes	Yes
2. Did the report of the review contain an explicit statement that the review methods were established prior to the conduct of the review and did the report justify any significant deviations from the protocol?	Yes	No
3. Did the review authors explain their selection of the study designs for inclusion in the review?	Yes	Yes
4. Did the review authors use a comprehensive literature search strategy?	Yes	Yes
5. Did the review authors perform study selection in duplicate?	Yes	Yes
6. Did the review authors perform data extraction in duplicate?	Yes	Yes
7. Did the review authors provide a list of excluded studies and justify the exclusions?	No	No
8. Did the review authors describe the included studies in adequate detail?	Yes	Yes
9. Did the review authors use a satisfactory technique for assessing the risk of bias (RoB) in individual studies that were included in the review?	Yes	Yes
10. Did the review authors report on the sources of funding for the studies included in the review?	Yes	No
11. If meta-analysis was performed did the review authors use appropriate methods for statistical combination of results?	Yes	Yes
12. If meta-analysis was performed, did the review authors assess the potential impact of risk of bias in individual studies on the results of the meta-analysis or other evidence synthesis?	Yes	Yes
13. Did the review authors account for RoB in individual studies when interpreting/discussing the results of the review?	Yes	Yes
14. Did the review authors provide a satisfactory explanation for, and discussion of, any heterogeneity observed in the results of the review?	Yes	Yes
15. If they performed quantitative synthesis did the review authors carry out an adequate investigation of publication bias (small study bias) and discuss its likely impact on the results of the review?	Yes	Yes
16. Did the review authors report any potential sources of conflict of interest, including any funding they received for conducting the review?	Yes	Yes
Result	High	High
Pass/Fail	Pass	Pass

**Table 4:** Results of the SANRA 2 assessment tool for narrative reviews by review authors Passing score: 9/12 SANRA: Scale for the Assessment of Narrative Review Articles

First author	Justification of the article’s importance for the readership	Statement of concrete aims or formulation of the question	Description of the literature search	Referencing	Scientific reasoning	Appropriate presentation of data	Sum	Pass/fail
Fishbein Abet al. [3]	2	2	1	1	2	1	9	Pass
Russell G. Foster et al.	0	0	0	2	1	1	4	Fail
Foster RG. Sleep et al. [4]	1	2	1	1	2	2	9	Pass
Janse van Rensburg DC et al. [5]	2	1	1	2	2	2	10	Pass
Lee Y et al. [6]	2	2	1	2	2	2	11	Pass
Li Y et al. [7]	2	2	2	2	2	2	12	Pass
Poza JJ et al. [8]	2	1	1	2	2	2	10	Pass
Senesi P et al. [9]	1	1	1	2	2	2	9	Pass
Spaleniak W et al. [10]	1	1	1	2	2	2	9	Pass
Sun SY et al. [11]	2	2	2	2	2	2	12	Pass
Drăgoi CM et al. [15]	2	2	1	1	2	1	9	Pass
Samanta S et al. [16]	2	1	1	2	2	2	10	Pass
Roenneberg T et al. [17]	1	2	1	2	2	2	10	Pass
Moreno CRC et al. [18]	2	2	1	1	2	1	9	Pass
Healy KL et al [19]	2	1	0	2	2	2	9	Pass
Lee Y et al. [20]	1	2	1	2	2	2	10	Pass
Aykan U et al. [21]	2	2	1	2	2	2	11	Pass
Vasey C et al. [22]	2	2	2	2	2	2	12	Pass
Yuan M et al. [23]	2	2	2	2	2	2	12	Pass
Breitenbach T et al.	0	1	0	2	2	1	8	Fail
Kramer A et al.	1	0	0	1	1	1	4	Fail
Cornelissen G et al.	1	1	1	2	2	2	9	Pass
Kallweit, M.S et al	2	2	1	2	2	2	11	Pass
Von Gall, C et al.	2	2	1	2	2	2	11	Pass

Discussion

Health Implications of Circadian Disruption

Circadian dysregulation, characterized by disturbances or loss of rhythmicity, has emerged as a significant contributor to various health issues, including cardiovascular, neurological, psychological, and reproductive disorders. Numerous studies have demonstrated that maintaining a regular circadian rhythm has a profound impact on cognitive and physical functioning, influencing performance variability. This variability tends to increase as the complexity of tasks rises. For example, Drăgoi CM et al. conducted a study using a 20-hour forced desynchrony protocol, which revealed peak performance in tasks such as psychomotor vigilance, short-term memory, calculations, digit symbol substitution, and alertness. These peaks coincided with the maximum core body temperature (CBT), just before the onset of melatonin secretion. Conversely, a significant decline in performance was observed at the lowest body temperature, occurring just after melatonin secretion peaked. The study also found that prolonged wakefulness impaired cognitive performance, with higher performance ratings typically recorded during the day and evening compared to night or early morning, further underscoring the role of circadian rhythms in optimizing activity performance [15]. In summary, maintaining a regular circadian rhythm is crucial for optimizing cognitive and physical performance, with disruptions leading to significant declines in both, particularly during night and early morning hours.

Mental Health Issues

Professions that involve frequent travel, such as airline crew members, and their associated shift work, can significantly impact and may tremendously increase the risk of mental health issues. This heightened risk is primarily due to night-time light exposure, which leads to the desynchronization of circadian rhythms. Yuan M et al. highlighted in their study that jet lag can exacerbate major depressive disorder and is also associated with mood changes, dysphoria, and bipolar disorder. The study further noted that eastward travel over an extended period, such as a month, can contribute to anxiety and depression. Regarding travel direction, traveling from east to west increases the risk of depression, while traveling from west to east may trigger manic episodes [16]. Moreover, staying awake at night which is commonly seen in pilots and aircrew members, is a known risk factor for relapse of substance use disorders and new or recurrent major depressive disorder [17]. Frequent travel and night-time light exposure, common in professions like airline crew, significantly heighten the risk of mental health issues, including but not limited to depression, mood disorders, and anxiety with travel direction playing a key role in exacerbating symptoms.

Other Systems

Circadian rhythm disturbances affect multiple systems, including the neurological and cardiovascular systems, metabolism, cancer progression, and fertility. Table 5 summarizes the impact of circadian disruption on these systems, highlighting the broad scope of its negative effects. This emphasizes the importance of maintaining circadian health to prevent long-term consequences and underscores the need for interventions that address these disruptions to promote overall well-being.

**Table 5:** Impact of circadian disruption on neurology, cardiovascular, metabolic health, cancer progression, and reproductive health IARC: International Agency for Research on Cancer

System	Impact of circadian disruption on the system
Neurology	Circadian disruption interferes with an individual at the neurological level as well. It leads to the formulation and accumulation of misfolded protein aggregates, which may trigger neurodegenerative diseases like Alzheimer’s disease. It also leads to the aging of the brain demonstrated by Yuan M et al. in their study that post-mortem tissue of patients with neurodegenerative disorders showed disruption in the rhythmic expression of circadian genes [16]
Cardiovascular system	The circadian rhythm regulates key cardiovascular functions, including the adrenal medullary system, the renin-angiotensin system, and sympathetic activity, which in turn influence catecholamine secretion and aldosterone biosynthesis. Disruption of the circadian rhythm can negatively impact these processes and alter fuel metabolism in the heart. Moreover, professions that involve night shifts, such as airline crew members, are at an increased risk of stroke, heart attacks, and other coronary events [16,17]
Metabolic disorders	Circadian disruptions play a significant role in metabolic health. These disruptions interfere with processes such as fat production and oxidation, leading to an increased risk of type 2 diabetes and obesity, particularly in individuals with irregular schedules [16]. The circadian system also regulates hormones like leptin and ghrelin, which are critical in controlling hunger and satiety [17]. The intersection between circadian biology and metabolic health presents an important area for future research, offering the potential for developing targeted interventions that could reduce the risk of metabolic disorders by aligning these processes with natural circadian rhythms
Circadian disruption and its link to cancer progression	Circadian misalignment is also linked to cancer progression, especially among shift workers who experience chronic disruptions. The IARC has classified shift work that disrupts circadian rhythms as potentially carcinogenic (Group 2A) [16]. This classification serves as a stark reminder of the serious health consequences associated with our increasingly 24/7 lifestyle. The long-term risks, including an elevated risk of cancer, are becoming increasingly clear as we continue to push the boundaries of natural circadian rhythms with artificial lighting and around-the-clock activities
Reproductive health	Additionally, reproductive health, particularly male fertility, is influenced by the regulation of hormone release through clock gene expression [16]. As concerns about reproductive health become more widespread, a deeper understanding of the role circadian rhythms play could lead to new strategies for preserving fertility and promoting overall reproductive health.

Jet Lag Syndrome

Jet lag syndrome occurs when rapid travel across time zones causes a misalignment of the circadian rhythm, resulting in various symptoms. In other words, it happens when the body’s internal clock falls out of sync with the local time after crossing multiple time zones. According to Janse van Rensburg DC et al., it typically takes about 0.5 days per time zone crossed when traveling westward (approximately two hours of adjustment per day) for natural circadian realignment. In contrast, eastward travel takes about one day per time zone crossed (adjusting by one hour per day) [5]. Until this realignment occurs, the circadian rhythm remains disrupted, affecting systems that depend on it, including cognitive and physical performance. Li Y et al. also found that eastward travel, which requires advancing the circadian phase, poses a greater challenge than westward travel, as our circadian clock is usually slightly longer than 24 hours, making it easier to delay than advance the internal clock [7].

The symptoms of jet lag vary from one individual to another but may include daytime fatigue, difficulty sleeping at night, reduced appetite, digestive discomfort, cognitive difficulties, and mood disturbances. For those who frequently travel or work irregular hours, such as flight attendants and night shift workers, the effects of chronic jet lag can extend beyond temporary inconvenience and contribute to serious long-term health issues.

In summary, jet lag is caused by the misalignment of the circadian clock due to rapid time zone changes, with eastward travel presenting greater challenges for circadian adjustment. This review will explore the best interventions to address circadian misalignment, such as light therapy, chrono-nutrition, and other methods. Effective management, particularly for frequent travelers and shift workers, is essential to mitigating the long-term health effects of chronic jet lag and ensuring smoother circadian transitions during travel. 

Chronotherapy

Chronotherapy is an effective way to manage circadian misalignment or any delayed sleep-wake phase disorder (DSWPD). It involves delaying bedtime until the desired sleep-wake cycle is achieved. For instance, an individual can delay bedtime or wake time by three hours every two to five days until the sleep-wake cycles align with the societal bedtime and wake-up hours. Numerous small studies have reported the success of this time-varying therapy, indicating it is well-tolerated by typical DSWPD patients [11]. Therefore, chronotherapy not only addresses sleep timing but also improves overall sleep quality and duration, making it a viable treatment for circadian rhythm disorders.

Concept of Sleep Hygiene

The concept of sleep hygiene dates back to the late 19th century, as reported in a study by Moreno CRC et al. It was introduced by Marie de Manacéïne in her work, "Sleep: Its Physiology, Hygiene, and Psychology." The concept was popularized in the late 1970s by Peter Hauri's book "Current Concepts: The Sleep Disorders." Sleep hygiene comprises recommendations aimed at promoting adequate and healthy sleep through good habits and practices. These practices as per Moreno CRC et al. include creating an environment that’s restful, maintaining a regular sleep schedule, and avoiding stimulants before bedtime [18]. Sleep hygiene, therefore, remains fundamental in managing sleep disorders and improving overall sleep health.

Chrono-Phototherapy

Chrono-phototherapy, which involves the use of bright light therapy, is an effective tool for alleviating motor disorders, sleep/wake disturbances, anxiety, and depression, particularly in patients with neurodegenerative diseases. Light exposure has a significant influence on melatonin levels, as melatonin production is suppressed by light. Experimental studies using simulated night shift models have shown that bright light can quickly adjust peripheral clocks, making it a promising non-pharmacological intervention to counter the negative effects of shift work and jet lag [6]. According to the study by Sun SY et al., light therapy can alter the phase, direction, and amplitude of the circadian rhythm depending on the time, intensity, spectral properties, and duration of the light exposure. Furthermore, studies have shown that morning light can be used to advance the circadian cycle, while evening or bedtime light can delay it [11].

Moreno CRC et al. have extensively discussed the properties of light and its impact on circadian alignment. Proper synchronization of circadian rhythms relies on variations in light intensity and spectrum. Sunlight, with its higher proportion of short wavelengths, has a greater impact on circadian rhythms compared to artificial light, making it the most effective source for aligning the circadian clock. To promote circadian alignment, particularly in urban areas, spending more time outdoors-especially in the morning when sunlight is rich in short wavelengths, is strongly recommended. Prolonged daytime exposure to bright sunlight can also mitigate the effects of nighttime artificial light by reducing the sensitivity of intrinsically photosensitive retinal ganglion cells (ipRGCs), which play a crucial role in melatonin suppression [18].

This demonstrates that circadian synchronization through light exposure is influenced by prior light exposure, or "photic history," making natural sunlight the best source for aligning the internal circadian clock with the external environment. This alignment promotes better sleep patterns and overall health.

Chrono-Diet

The chrono-diet principle involves restricting food intake to specific daily intervals, known as time-restricted feeding (TRF) or time-restricted eating (TRE) in humans, and this has become a popular strategy to synchronize peripheral clocks and improve metabolic health. The dynamic interaction between circadian clocks and metabolic pathways is increasingly recognized, as the circadian clock synchronizes diverse metabolic processes such as gluconeogenesis, mitochondrial metabolism, and lipogenesis [6]. 

As per Healy KL et al., meal timing does not entrain the SCN (a part of the brain that is responsible for circadian rhythm) but serves as a strong zeitgeber for peripheral clocks, coordinating circadian timing with feeding and fasting [16]. A strong circadian alignment is required to balance anabolism and catabolism. Nutrient sensors such as Sirtuin 1 (SIRT1), poly (ADP-ribose) polymerase-1 (PARP-1), AMP-activated kinase (AMPK), and mechanistic target of rapamycin (mTOR) regulate metabolic pathways and modulate circadian clock function [19]. Hence, short-term feeding at the wrong time can desynchronize peripheral clocks, leading to obesity, hyperphagia, physical inactivity, and metabolic disorders [20].

Chrono-diet, focusing on TRF or TRE, supports circadian alignment by synchronizing mealtimes with the body's natural biological clocks. This alignment not only maintains overall circadian rhythm stability but also focuses on reducing the risk of metabolic disorders.

Chrono-Exercise

Exercise also plays a significant role as a non-photic stimulus that can influence the entrainment of circadian clocks. Studies suggest that based on individual chronotypes, exercise can induce phase-shifting effects (advance or delay). For example, when compared to sedentary males, male rugby players have a higher average expression of core-clock genes [15]. Studies as mentioned by Moreno CRC et al. have demonstrated that nocturnal physical exercise under constant conditions can cause significant phase delays in circadian hormonal rhythms [18].

Body temperature, which affects physical exercise performance, exhibits circadian variation, generally peaking in the late afternoon. This variation can impact performance, with a greater tendency to dissipate body heat in the afternoon explaining better exercise performance at that time. However, the timing of exercise should consider individual circadian phenotypes, with light and moderate exercises in the morning being beneficial for morning types [18]. Exercise is a potent factor for resetting both central and peripheral clocks, including skeletal muscle clocks, through activity-dependent expression of core clock genes. Timing of physical activity is crucial for regulating the circadian clock, with early-evening exercise causing significant phase advances and late-night exercise causing phase delays [19].

Chrono-exercise, which involves timing physical activity to align with circadian rhythms, enhances circadian alignment by optimizing the body's natural energy cycles. Exercising at specific times of the day can reinforce circadian stability, improving both physical performance and overall metabolic health.

Melatonin

Melatonin, a hormone produced by the pineal gland, is synthesized from serotonin. The endogenous clock situated in the suprachiasmatic nucleus (SCN) of the hypothalamus governs the rhythm of melatonin secretion, as well as other circadian rhythms in mammals, including feeding patterns and the sleep-wake cycle. Melatonin is known to have both preventive and therapeutic anticancer properties. Its antioxidative mechanisms have been observed to decrease the toxicity of harmful drugs, particularly neurotoxic agents like methamphetamine. Additionally, pineal melatonin plays a role in perpetuating oxidative and inflammatory processes, which contribute to the development of various medical conditions such as cancer and neurodegenerative disorders [21].

The release of melatonin and the regulation of circadian rhythm are strongly associated with multiple neurotransmitter systems within the central nervous system (CNS). The melatonergic system can be influenced by neurotransmitters including serotonin, dopamine, norepinephrine, and histamine, which can have both stimulatory and inhibitory effects. Each of these pathways presents potential pharmaceutical targets for treating numerous diseases that are centrally mediated.

Jet lag syndrome, resulting from the misalignment of the internal circadian clock due to rapid transmeridian travel, leads to symptoms like fatigue, insomnia, cognitive distress, and digestive issues. Melatonin helps resynchronize the circadian rhythms disrupted by jet lag, thereby promoting better sleep-wake cycles. The hormone's interactions with neurotransmitter systems such as serotonin, dopamine, norepinephrine, and histamine highlight its therapeutic potential. Combining personalized light therapy, which adjusts exposure based on an individual's circadian phase, with melatonin supplementation, offers an effective non-pharmacological approach to alleviate the symptoms of jet lag and restore circadian harmony [22].

To summarize, melatonin supplementation supports circadian alignment by helping to resynchronize the body's internal clock during jet lag. By mimicking the body's natural melatonin release, it can reduce the severity of jet lag symptoms, promoting faster adjustment to new time zones and improving sleep quality.

Electroacupuncture

Electroacupuncture, a treatment approach in traditional Chinese medicine, has shown positive regulatory effects on circadian rhythms. It promotes rhythm resynchronization in an advanced light-dark cycle-shifting environment, with varying effects depending on the time of application. Electroacupuncture significantly promotes rhythm resynchronization at specific times, such as ZT4, ZT8, and ZT12, and can regulate and restore the rhythm of melatonin secretion, thereby improving insomnia in rat models [23]. This technique highlights the potential of electroacupuncture in managing circadian rhythm disruptions and associated sleep disorders.

Modulating Casein Kinases

Familial advanced sleep phase syndrome (FASPS) is caused by a T44A mutation in CK1δ that leads to hypophosphorylation of PER2 and shortens the circadian period. CK1δ, despite being a ubiquitous protein required for many functions, is a crucial component of the circadian clock. Meng et al. demonstrated that pharmacological inhibition of CK1δ by PF-670462, a selective CK1δ inhibitor, restores robust and persistent circadian rhythms in vipr2−/− mice, which have disrupted rhythms due to the loss of an SCN signal, vasoactive intestinal peptide receptor type 2 [6]. Thus, modulating CK activity helps restore circadian rhythm stability by adjusting the phosphorylation of clock proteins, offering a potential therapeutic approach for correcting circadian misalignments.

Modulating Nuclear Receptors

Lee Y et al. study shows that compounds that act as REV-ERB (nuclear receptors) agonists, such as SR9009 and SR9011, have been shown to induce wakefulness, suppress sleep, regulate emotional behavior in mice, and reduce anxiety-like behavior [6]. These findings highlight the potential of targeting REV-ERBs in regulating sleep-wake cycles and emotional behaviors.

Chrono-Modulation With Natural Compounds

Natural compounds can also modulate circadian clock function. Resveratrol, a potent natural activator of the NAD^+^-dependent deacetylase SIRT1, targets PER2 and restores rhythms in clock gene expression, improving lipid metabolism in HFD-induced obese mice. Nobiletin (NOB), a dietary flavonoid found in citrus fruits, acts as a RORα and RORγ agonist, enhancing circadian rhythms and protecting against metabolic syndrome [6]. Additionally, Spaleniak W et al. study on resveratrol has shown promise in various health issues, including obesity, diabetes, cancer, cardiovascular diseases, and neurodegenerative diseases. Its ability to activate SIRT1 and modulate signaling pathways suggests significant potential in normalizing circadian rhythms and mitigating age-related sleep disturbances [10]. Hence, chrono-modulation with natural compounds, such as resveratrol and NOB, can aid in mitigating jet lag by supporting the realignment of circadian rhythms, enhancing adaptation to new time zones, and reducing related symptoms.

Cognitive Behavioral Therapy and Other Treatments

Cognitive Behavioral Therapy (CBT) for Insomnia (CBT-I) can be particularly effective in managing the psychological aspects of jet lag syndrome, such as anxiety related to sleep disturbances during travel. Additionally, strategies like stress management techniques and relaxation exercises may help reduce the cognitive burden associated with disrupted sleep patterns. These approaches address the mental and emotional challenges of adjusting to new time zones, complementing other physiological interventions for jet lag (11). 

Incorporating CBT-I and stress management techniques into jet lag management plans provides a comprehensive approach that not only addresses the physical symptoms but also the psychological discomfort associated with disrupted sleep patterns. By focusing on both the mind and body, these strategies can enhance overall adaptation to new time zones, making travel experiences more manageable and less disruptive to daily life. A summary of these interventions and their impact on regulating the circadian rhythm is presented in Table 6.

**Table 6:** Summary of interventions for regulating circadian rhythm and their impact CBT: cognitive behavioral therapy; CK: casein kinase; NOB: nobiletin

Intervention	Summary
Chronotherapy	Chronotherapy involves gradually delaying bedtime to align the sleep-wake cycle with societal norms, improving sleep quality and duration.
Sleep hygiene	Sleep hygiene focuses on healthy sleep habits, such as maintaining a regular schedule and creating a restful environment, to improve overall sleep health.
Chrono-phototherapy	Bright light therapy shifts circadian phases and regulates melatonin levels, offering an effective non-pharmacological intervention for sleep and mood disorders.
Chrono-diet	Chrono-diet synchronizes mealtimes with circadian rhythms, improving metabolic health and reducing the risk of disorders like obesity and type 2 diabetes.
Chrono-exercise	Chrono-exercise aligns physical activity with circadian rhythms to enhance performance, energy cycles, and metabolic health, optimizing circadian stability.
Melatonin	Melatonin supplementation resynchronizes circadian rhythms, alleviating jet lag, and improving sleep-wake cycles by mimicking natural melatonin release
Electroacupuncture	Electroacupuncture promotes circadian resynchronization and melatonin regulation, helping manage sleep disorders, and circadian misalignments
Modulating CKs	Modulating CK activity restores circadian stability by regulating clock proteins, providing a therapeutic approach for circadian rhythm disorders
Modulating REV-ERBs	REV-ERB agonists regulate sleep-wake cycles and emotional behavior, offering potential treatment for sleep and mood disorders
Chrono-modulation with natural compounds	Natural compounds like resveratrol and NOB enhance circadian rhythms, supporting metabolic health, and mitigating jet lag symptoms
CBT	CBT-I addresses psychological aspects of jet lag by managing anxiety and sleep disturbances, complementing physiological treatments

## Conclusions

Jet lag syndrome, caused by the misalignment of the body's internal clock due to rapid time zone changes, leads to symptoms like fatigue, insomnia, and cognitive distress. Addressing these disruptions is crucial to prevent long-term health issues such as mental disorders, cardiovascular diseases, and metabolic problems. Among various interventions, melatonin supplementation and personalized light therapy are effective in realigning circadian rhythms and reducing jet lag severity. Complementary strategies like chrono-diet, chrono-exercise, and electroacupuncture further support circadian alignment, enhancing overall health and well-being during travel. A comprehensive, personalized approach integrating these methods can significantly improve adaptation to new time zones and mitigate the negative effects of jet lag. Future research should explore the long-term efficacy and broader applications of these interventions.
